# The Achene Mucilage Hydrated in Desert Dew Assists Seed Cells in Maintaining DNA Integrity: Adaptive Strategy of Desert Plant *Artemisia sphaerocephala*

**DOI:** 10.1371/journal.pone.0024346

**Published:** 2011-09-02

**Authors:** Xuejun Yang, Wenhao Zhang, Ming Dong, Ivan Boubriak, Zhenying Huang

**Affiliations:** 1 State Key Laboratory of Vegetation and Environmental Change, Institute of Botany, Chinese Academy of Sciences, Beijing, China; 2 Graduate University of Chinese Academy of Sciences, Beijing, China; 3 Department of Biochemistry, Oxford University, Oxford, United Kingdom; 4 Institute of Cell Biology and Genetic Engineering, Kiev, Ukraine; Universita' di Milano, Italy

## Abstract

Despite proposed ecological importance of mucilage in seed dispersal, germination and seedling establishment, little is known about the role of mucilage in seed pre-germination processes. Here we investigated the role of mucilage in assisting achene cells to repair DNA damage during dew deposition in the desert. *Artemisia sphaerocephala* achenes were first treated γ-irradiation to induce DNA damage, and then they were repaired *in situ* in the desert dew. Dew deposition duration can be as long as 421 min in early mornings. Intact achenes absorbed more water than demucilaged achenes during dew deposition and also carried water for longer time following sunrise. After 4-d dew treatment, DNA damage of irradiated intact and demucilaged achenes was reduced to 24.38% and 46.84%, respectively. The irradiated intact achenes exhibited much higher DNA repair ratio than irradiated demucilaged achenes. Irradiated intact achenes showed an improved germination and decreased nonviable achenes after dew treatment, and significant differences in viability between the two types of achenes were detected after 1020 min of dew treatment. Achene mucilage presumably plays an ecologically important role in the life cycle of *A. sphaerocephala* by aiding DNA repair of achene cells in genomic-stressful habitats.

## Introduction

Plants have efficient adaptive systems to help them survive in different ecological environments. In the desert environment, environmental conditions such as precipitation are extremely fluctuating and unpredictable [1], [2]. As desert plants are exposed to such stressful environments, they have evolved numerous physiological and morphological traits to adapt to the harsh conditions. In addition, adaptation is a complex process in which populations of organisms can respond to long-term environmental stresses by permanent genetic change [3]. For those plants growing in the moving and semi-stable sand dunes, such molecular mechanisms are of vital importance for their long-term survival strategy that involves survival on or near the surface of dry and torrid sands.

It is essential for an organism to protect its DNA integrity since the genome contains all the information required for its development and reproduction. Meanwhile, DNA integrity of the living cells is constantly challenged by various genotoxicities, originating from either exogenous (such as UV light, irradiation and ozone) or endogenous (such as replication errors and oxidative byproducts of cellular metabolism) sources [4]–[7]. Thus, faithful and effective repair of DNA damage is of equal importance to prevention of DNA damage for the maintenance of genome integrity [8], [9]. DNA damage can cause chromosome rearrangements, such as reciprocal translocation, insertions, inversions, duplications and deletions, thus leading to shortening of lifespan [10], [11]. During their evolution, organisms have developed multiple systems to repair damage of irreversible mutations over their lifespan [9]. The gene products involved in these repair pathways are found to be evolutionarily conserved [11]. Once DNA damage occurs, different repair pathways are initiated according to the chemical and physical properties of the damage [12], [13]. The efficient repair of DNA damage will contribute to the maintenance of genomic integrity of organisms.

The single cell gel electrophoresis (comet) assay has long been used to investigate the DNA repair of genomic DNA in the studies of DNA repair kinetics and successfully applied to study DNA repair in a number of plant species as well as irradiated seeds [14]–[16]. During electrophoresis, DNA fragments migrate out of the cells to form a tail (comet) towards the anode [17], [18], and the unbiased detection of DNA damage by this method is superior to non-direct methods of DNA damage quantification [19], [20].

Plants are frequently exposed to environmental genotoxicities that target their genome integrity because of their sedentary nature and need for sunlight [6], [21]. These genotoxicities can lead directly or indirectly via generation of reactive oxidative species (ROS) to DNA lesions including apurinisations, single strand breaks (SSBs) and double-strand breaks (DSBs), the latter being most harmful because they can lead to major karyotypic instability and cell death [20]. Thus, plants are equipped with efficient systems to repair frequently occurring DNA damage. In seeds, previous studies have shown that cells have the potential to immediately repair DNA damage when rehydration takes place and that a functional DNA repair system is essential for seed survival [8], [22], [23]. DNA repair takes place in the first phase of rehydration when the embryo cells are still at G1 stage before DNA replication [24], [25]. The embryos of rye (*Secale cereale*) seeds, for instance, can repair DNA damage evoked by γ-irradiation after the embryo has become fully hydrated [25]. Therefore, hydration is a requisite not only for germination but also for metabolic re-activation, i.e. if the water potential is too negative to initiate full germination, seeds can still rapidly resume metabolic activities (including DNA repair), which are crucial for maintaining their viability [24], [26]–[28]. Although the DNA repair in plants and seed cells has been well studied under the laboratory or greenhouse conditions, there is extremely scarce evidence for this molecular mechanism from field conditions that plants actually experience.

Upon imbibition of water, seeds of many species release pectinaceous mucilage (known as myxospermy). The mucilage has been shown to play an important role in supporting seed dispersal and germination by the following mechanisms. Firstly, the mucilage retains moisture for seeds during germination and early seedling growth [29]–[31], and increases the moisture supply to the seed and minimizes water loss by enlarging the area of contact of seed with soil [32]–[34]. Secondly, the mucilage forms strong adherence to the soil surface once the mucilage is dehydrated, thus preventing further dispersal of the seed by rain and wind and of collection by ants or other seed predators [35]–[37]. Thirdly, the mucilage initiates or enhances seed germination by supplying water [32], [33], [38], [39]. Fourthly, the mucilage of some species like *Cavanillesia platanifolia* permits seeds in different stages of development within fruits to be fully mature until the beginning of rainy season [31], [40]. Finally, the mucilage aids seed germination in osmotically stressful and saline habitats of the cold desert environment [41]. However, little is known about the roles of the mucilaginous layer in seed pre-germination processes, especially in those associated with biochemical and molecular ones.

Seeds of many annuals and shrubs in desert regions remain on or near the surface of dry sands during or after dispersal, where they are exposed to intensive genotoxic agents, e.g. UV irradiation, high temperature and water deficit. To maintain their genome integrity, they have evolved special DNA repair mechanisms as part of their long-term survival strategy. Our previous study has indicated that mucilage formed by wetting the achenes with dew at night may enable the embryo to repair DNA, thus helping to maintain its viability under the harsh desert conditions [42]. However, neither the DNA damage before dew treatment nor DNA repair after dew treatment was quantitatively assessed in the previous study. Also, how the DNA repair affects seed viability and germination remains unknown. In this work, the role of mucilage in assisting of achene cells to repair DNA damage was evaluated by treating the achenes *in situ* with desert dew. To elucidate the molecular mechanisms by which desert plants maintain genome integrity, we further described the DNA repair kinetics of the achene cells in a quantitative manner. More specifically, we addressed the following questions: (1) can mucilage help the hydration of *A. sphaerocephala* achenes during dew deposition in the desert? (2) can this hydration promote the repair of damaged DNA in achene cells? and (3) can this repair mechanism be beneficial for maintaining achene germination and/or viability?

## Results

### Dew deposition

**Figure 1:**
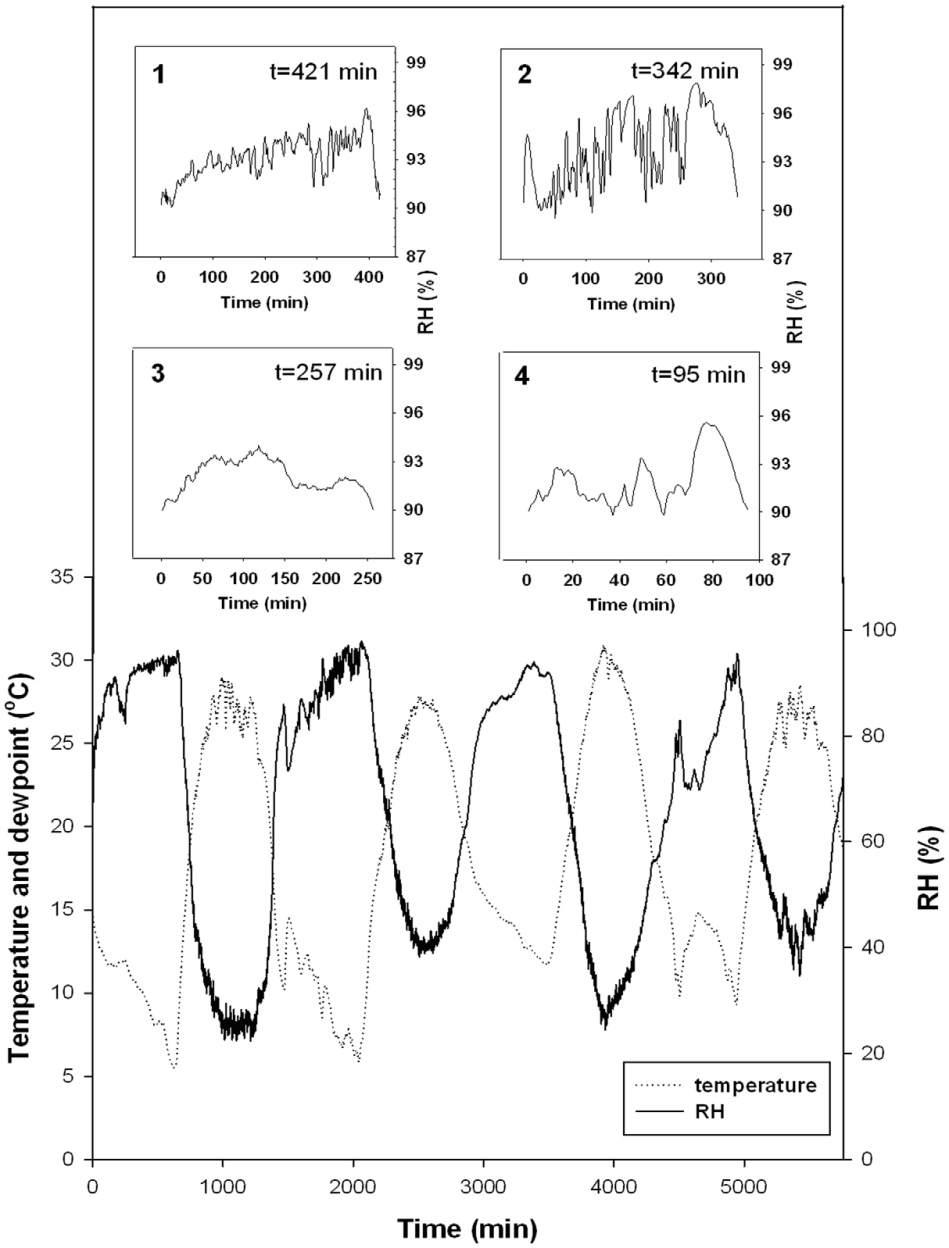
Temperature and RH during dew experiment (from 20:00 pm 28 August to 20:00 pm 1 September, 2009). Insets show the details of RH kinetics during dew deposition in early morning of each day, and dew deposition durations (t) of each day are indicated. Inset 1 was 29 August, inset 2 30 August, inset 3 31 August and inset 4 1 September.

**Figure 2:**
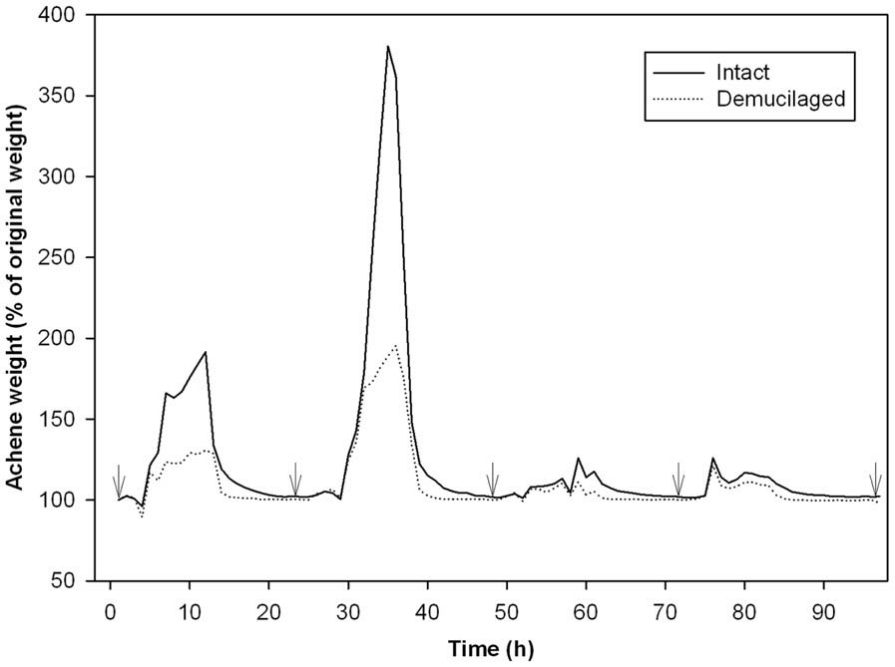
Weights (% of their original weight) of intact and demucilaged *A. sphaerocephala* achenes during dew treatment (from 20:00 pm 28 August to 20:00 pm 1 September, 2009). Achenes were weighed at 1 h intervals, and achene weights were expressed as % of their original weight. All data were the mean of three replicates. The arrows indicate sampling times of achenes (at 20:00 pm each day).

To investigate the DNA repair of irradiated achenes, a dew experiment was carried out *in situ* in *A. sphaerocephala* natural habitats in desert. Throughout the dew experiment, temperature and relative humidity (RH) were recorded at 1 min intervals in a continuous manner (Figure 1). Dew depositions occurred in early morning with low temperature and high RH. *A. sphaerocephala* achenes absorbed water from air in early mornings when RH was above 90% (Figures 1 and 2), and the time period of RH above 90% was therefore consider as dew deposition time.

Dew deposition occurred in all mornings approximately from 1:00 am to 7:00 am during the dew experiment. Dew deposition period ranged from 95 to 421 min per night and was different for the four early mornings (Figure 1 1–4 ). The longest (421 min) and shortest (95 min) dew deposition time occurred in early morning of 29 August and 1 September, respectively. Dew deposition time was 342 min and 257 min in early morning on 30 August and 31 August. Therefore, 421, 763 (421+342), 1020 (421+342+257) and 1115 (421+342+257+95) min accumulative dew time were recorded after 1, 2, 3 and 4 nights of dew treatment, respectively. These results indicate that dew is abundant in the early morning in summer in the deserts. Following sunrise, RH gradually decreased due to the rising temperature. At mid-day, RH reached a minimum concurrent with the occurrence of the highest temperature. The maximal temperature at mid-day can be as high as 30°C, and the minimal RH can be less than 20% at the same time.

### Achene hydration and dehydration

Water hydration and dehydration of intact and demucilaged achenes were determined by weighing intact and demucilaged achenes every 1 h throughout the whole experimental period. Achenes absorbed water, and their weights increased in the early mornings as the result of dew deposition, but the water absorbed by achenes was gradually lost in the following sunrise due to a decrease in RH (Figure 2).

Weights of intact achenes were significantly higher than those of demucilaged achenes during dew deposition in all early mornings. Intact achenes increased to 191.56% of their initial weigh at 7:00 am 29 August, while demucilaged achenes increased to only 130.80% at the same time. At 6:00 am 30 August, intact achenes reached to their highest weight (380.62%) and was significantly higher than that of demucilaged achenes (188.82%; *P*<0.001). Moreover, weight losses of intact achenes took longer time than demucilaged achenes following sunrise. For example, after increased to their highest weight during dew deposition at 6:00 am 30 August, intact achenes took 7 h to return to less than 110% of their original weight, whereas demucilaged achenes took only 4 h to do so (Figure 2).

### DNA damage of achene cells treated with irradiation

**Figure 3:**
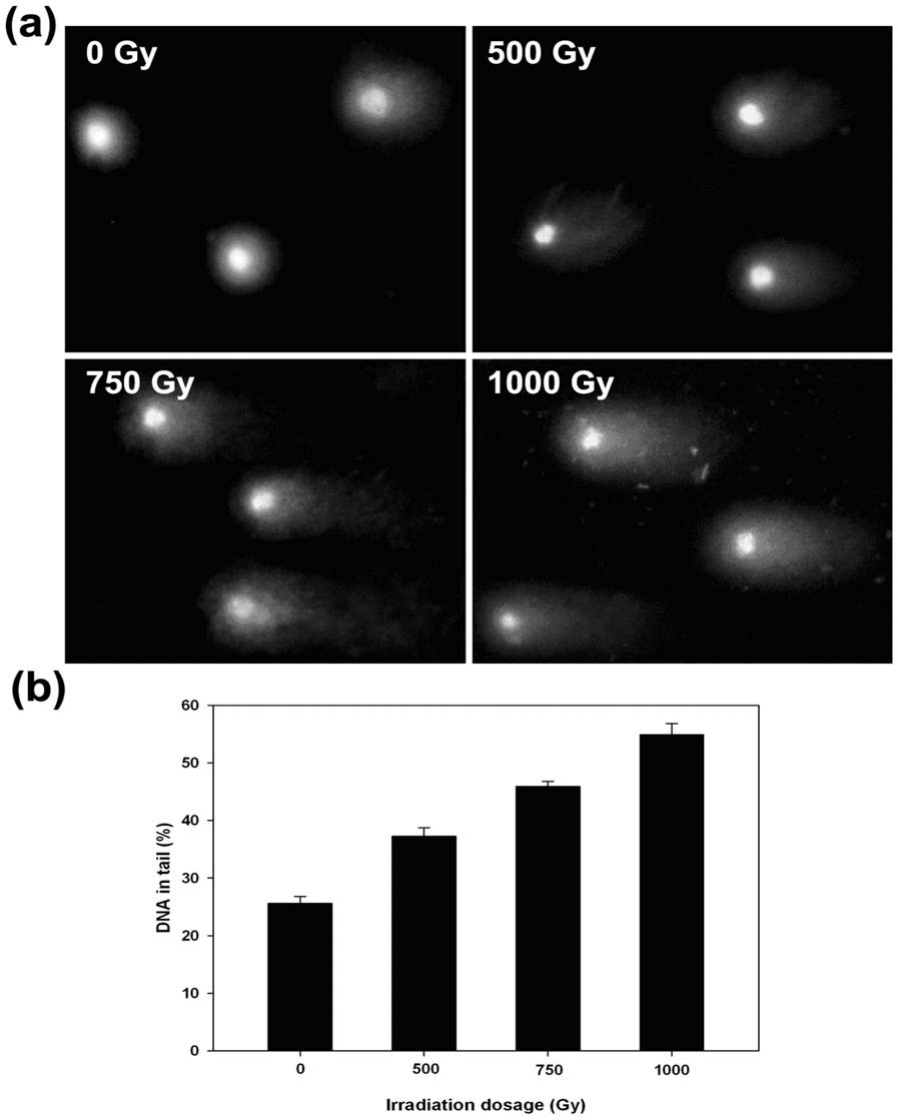
DNA damage of *A. sphaerocephala* achenes treated with different doses of γ-irradiation detected by comet assay. (a) showing representative comets of intact achenes treated with 0 (control), 500, 750 and 1000 Gy of irradiation. (b) showing %DNA in tail for each irradiation dose. Mean values were three replicates of 60 comets each. Error bars represent s.e.

To introduce DNA damage to achene cells, *A. sphaerocephala* achenes were treated with different doses of γ-irradiation. After irradiation, the comet assay showed that DNA damage of the achenes increased with elevated irradiation dose (Figure 3a). Control achenes (0 Gy) had little DNA fragments in comet tails, and more DNA fragments migrated to comet tails when irradiation dose increased (Figure 3a). Analysis of DNA percentage in tails in achene cells revealed significant difference among irradiation doses (*F* = 70.647, *P*<0.001). Further analysis showed that there existed a linear relationship between DNA percentage in tail and irradiation dose (*r^2^* = 0.972, *P*<0.001; Figure 3b). These results confirm that γ-irradiation is an effective method for evoking DNA damage to achene cells and that the comet assay is a valid method to measure DNA damage of achene cells in this study.

### DNA repair of achene cells

**Figure 4:**
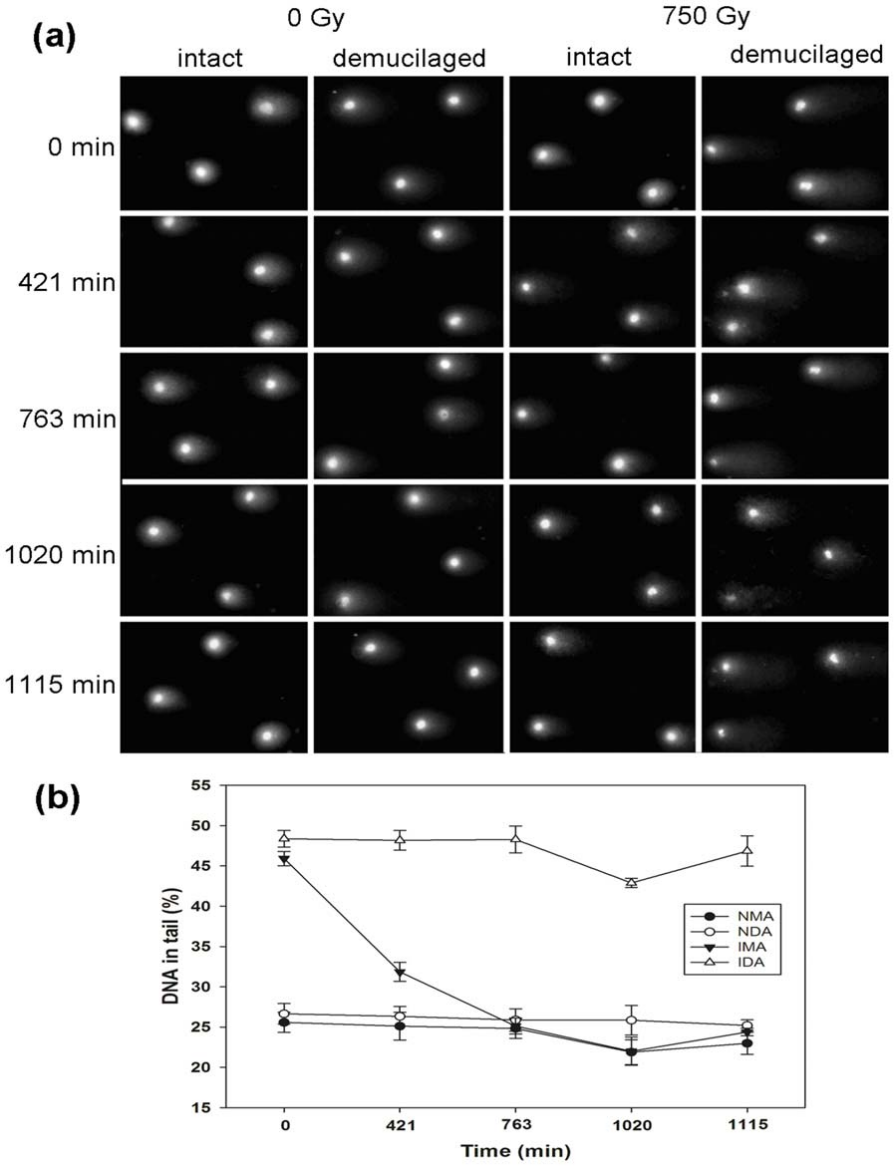
DNA damage of *A. sphaerocephala* achenes during 4 d dew experiments (from 20:00 pm 28 August to 20:00 pm 1 September, 2009), showing (a) representative comets of control intact and demucilaged achenes (0 Gy) and those of irradiated intact and demucilaged achenes (750 Gy) during dew treatment and (b) %DNA in tail for control intact and demucilaged achenes and those of irradiated intact and demucilaged achenes. NMA, non-irradiated intact achenes (control); NDA, non-irradiated demucilaged achenes; IMA, intact achene irradiated with 750 Gy; IDA, demucilaged achene irradiated with 750 Gy. Mean values were three replicates of 60 comets each. Error bars represent s.e.

In dew experiment, intact and demucilaged achenes of both control (NMA and NDA) and irradiated (IMA and IDA) treatments were sampled every 24 h, and DNA damage was detected by comet assay (Figure 4a, b). DNA damage of NMA did not obviously vary during dew treatment (*F* = 1.214, *P* = 0.364), a similar result was observed for the NDA (*F* = 0.169, *P* = 0.949; Figure 4a, b). No obvious decrease in DNA damage for IDA was observed (*F* = 2.920, *P* = 0.077). In contrast, an obvious reduction in DNA damage was observed in IMA (*F* = 76.093, *P*<0.001; Figure 4a, b). DNA damage in IMA was repaired to the level of control achenes after 763 min of dew exposure. After 1115 min (4 d) dew exposure, DNA damage of IDA and IMA was reduced to 46.85±1.89% and 24.38±0.47%, respectively (Figure 4b).

To further demonstrate DNA repair kinetics, DNA repair ratio was calculated for the four treatments at different dew treatment times (Table 1). In non-irradiated control achenes (NMA and NDA), DNA repair ratios of the cells were low during dew treatment, and no significant difference in DNA repair ratio was observed between the two treatments (Table 1). However, two-way ANOVA revealed that mucilage (*F* = 417.305, *P*<0.001), dew treatment time (*F* = 47.026, *P*<0.001) and their interactions (*F* = 29.212, *P*<0.001) had significant effects on DNA repair ratios of irradiated achenes. Similar to control achenes, IDA exhibited low DNA repair ratio, with the highest DNA repair ratio at 1020 min dew treatment. By contrast, IMA exhibited much higher DNA repair ratio than IDA, and t-test showed significant differences between the two types of irradiated achenes from the first dew treatment (Table 1). It was noted that DNA repair ratios of intact and demucilaged achenes all decreased after 1020 min dew exposure, which may resulted from the shortness of dew deposition (Figure 1) on that day or some systematic shift in the measurements for that day. These results indicate that the presence of mucilage had a significant positive effect on the DNA repair ratio of irradiated achenes.

**Table 1:** DNA repair ratios (%) of control intact and demucilaged achenes (0 Gy) and those of irradiated intact and demucilaged achenes (750 Gy) during dew treatment.

Dew time(min)	0 Gy		750 Gy	
	Intact	Demucilaged	Intact	Demucilaged
0	0^Aa^	0 ^Aa^	0^Aa^	0^Aa^
421	1.85±6.70^Aa^	2.04±4.51^Aa^	30.63±2.55^Ba^	0.38±2.42^Ab^
763	2.97±4.74^Aa^	0.87±4.74^Aa^	45.30±2.18^Ca^	0.17±3.32^Ab^
1020	14.43±6.05^Aa^	3.83±6.83^Aa^	52.09±3.82^Ca^	10.88±1.11^Bb^
1115	10.17±5.29^Aa^	6.24±2.56^Aa^	46.91±1.02^Ca^	3.02±3.76^ABb^

Different uppercase letters indicate significant differences between time of dew treatment for an achene type within an irradiation dosage and different lowercase letters indicate significant differences between intact and demucilaged achenes in an irradiation dose at a given dew treatment time (*P*<0.05).

### Achene germination and viability

Germination percentage and percentages of viable ungerminated and nonviable achenes of NMA did not significantly differ from those of NDA. In addition, there was no trend in improvement of germination and viability for achenes after dew treatment (Table 2). However, IMA showed an improved germination after dew treatment, and this improvement became significant (*P* = 0.048) after 1115 min of dew treatment. Moreover, percentage of nonviable achenes was significantly decreased after dew treatment in IMA, and there were significant differences between IMA and IDA after dew treatment for 1020 min (Table 2). These results suggest that achene mucilage hydrated at dew deposition can improve germination and viability of irradiated intact achenes. Meanwhile, percentage of nonviable achenes in IDA was also significantly decreased to 11% after 421 min of dew exposure, but it subsequently increased again from 1020 min of dew exposure. This may result from that the dense dew in first two mornings also possibly allows demucilaged achenes to restore viability, but such an effect would be insufficient in a condition where less dew is deposited.

**Table 2:** Germination and viability of control intact and demucilaged achenes (0 Gy) and those of irradiated intact and demucilaged achenes (750 Gy) of *A. sphaerocephala* after dew treatment.

Dew time (min)	0 Gy						750 Gy					
	Germination percentage(%)		Viable ungerminated(%)		Nonviable(%)		Germination percentage (%)		Viable ungerminated(%)		Nonviable(%)	
	Intact	Demucilaged	Intact	Demucilaged	Intact	Demucilaged	Intact	Demucilaged	Intact	Demucilaged	Intact	Demucilaged
0	89±6^Aa^	83±4^Aa^	9±5^Ba^	15±4^Ba^	1±1^Aa^	3±1^Aa^	55±1^Aa^	49±3^Aa^	24±4^Aa^	23±3^Aa^	21±3^Aa^	28±5^Aa^
421	71±7^Ba^	57±6^Ba^	27±7^Aa^	40±7^Aa^	3±1^Aa^	3±1^Aa^	67±7^ABa^	63±15^Aa^	24±6^Aa^	27±11^Aa^	9±4^Ba^	11±5^Ba^
763	80±4^ABa^	71±7^ABa^	20±4^ABa^	28±6^ABa^	0±0^Aa^	1±1^Aa^	65±3^ABa^	55±7^Aa^	28±5^Aa^	35±10^Aa^	7±3^Ba^	11±3^Ba^
1020	84±2^ABa^	76±5^Aa^	16±2^ABa^	20±5^Ba^	0±0^Aa^	4±0^Aa^	69±7^ABa^	61±7^Aa^	27±5^Aa^	19±6^Aa^	4±2^Ba^	20±2^ABb^
1115	81±5^ABa^	81±4^Aa^	16±4^ABa^	16±2^Ba^	3±1^Aa^	3±1^Aa^	71±1^Ba^	61±5^Aa^	27±1^Aa^	17±7^Aa^	3±1^Ba^	21±3^ABb^

Different uppercase letters indicate significant differences between time of dew treatment for an achene type within a germination category and different lowercase letters indicate significant differences between intact and demucilaged achenes in an irradiation dose at a given dew treatment time (*P*<0.05).

Three-way ANOVA showed that irradiation, mucilage and their interaction had significant effects on germination percentage but not dew treatment time and other interactions. Dew treatment time significantly affected the percentage of viable ungerminated achenes. In contrast, other factors and their interactions had no effect on the percentage of viable ungerminated achenes. Futhermore, irradiation, mucilage, dew treatment time and their interactions except for the interaction of three factors all had significant effects on percentage of nonviable achenes (Table 3).

**Table 3:** Three-way ANOVA of effects of mucilage, dew time, irradiation and their interactions on germination and viability of *A. sphaerocephala* achenes.

Source	Germination		Viable ungerminated		Nonviable	
	*F*-value	*P*-value	*F*-value	*P*-value	*F*-value	*P*-value
Mucilage (M)	7.433	**0.009**	0.781	0.382	25.621	**<0.001**
Dew time (T)	1.384	0.257	3.564	**0.014**	7.916	**<0.001**
Irradiation (I)	33.109	**<0.001**	2.742	0.106	117.835	**<0.001**
M× T	0.107	0.979	1.000	0.419	3.050	**0.028**
M× I	0.268	0.607	2.375	0.131	14.522	**<0.001**
T× I	4.613	**0.004**	1.642	0.183	7.358	**<0.001**
M×T× I	0.371	0.828	0.145	0.964	2.341	0.071

## Discussion

During their evolution, various plant species have developed different strategies and mechanisms to acclimate to their environments. For many species of Asteraceae, Brassicaceae, Lamiaceae, Plantaginaceae and other families that frequently occur in desert habitats, their seeds or fruits commonly develop an external mucilage layer, which is believed to be ecologically important for them to inhabit the harsh desert conditions [2], [37], [41]. In the present study, intact achenes of *A. sphaerocephala*, which absorb more water and retain longer hydration time, exhibited higher capacity to repair their DNA damage and restore DNA integrity than demucilaged achenes. Therefore, the mucilage of *A. sphaerocephala* achenes is expected to play an ecological role in DNA repair of achene cells during dew deposition in harsh desert environments.

In our study, DNA damage of the achene cells was quantitatively measured by using the comet assay, thus enabling DNA repair of the achene cells to be accurately estimated during dew treatment. Moreover, DNA damage of the achene cells was found to be linearly increased with irradiation dose (Figure 3), which is consistent with a previous analysis that the DNA damage of HT1080 cells increases linearly with increasing γ-irradiation dose [43]. This result indicates that achene cells of *A. sphaerocephala*, similar to the cells of other organisms, are vulnerable to irradiation.

Dew deposition occurred in early mornings throughout our experimental period, and dew deposition period can be as long as 421 min in the early morning (Figure 1). These dew deposition times were similar to those of previously reported in the Negev Desert (50–340 min per night; [42]). These findings indicate dew deposition is abundant in the desert conditions. Furthermore, it has been suggested that dew serves as an important water source for growth and reproduction of plants, biological crusts, insects and small animals in the harsh arid environments [44]–[46]. In the Negev Desert, for instance, the annual dew deposition is 33 mm with a mean of 195 dewy nights per year [47]. In addition, there are reports showing that dew can be an important source of water for the growth and development of reproductive organs of *Bryum dunense* [48], [49]. In Mu Us Sandland, the daily dew deposition can be as high as 0.079 to 0.206 mm d^−1^ and can also serve as a water source for growth and development of the organisms in this region [45], [46]. Therefore, it is conceivable that the daily dew deposition in Mu Us Sandland can be used to repair DNA damage of living cells.

The weights of intact achenes were significantly higher than demucilaged achenes during dew deposition periods in all early mornings (Figure 2), implying that more water is likely to be absorbed by intact achenes during dew deposition. This result further confirms the previous report that intact achenes have higher water-carrying capacity [42]. As found in *Artemisia monosperma* [50], our results also reveal a higher water-absorbing capacity for myxospermy in the hydrated condition. In the desert environment, dew deposited in the soil surface would evaporate rapidly following sunrise [44]. In our study, dew absorbed by achenes also evaporated very quickly following sunrise, but weight losses of intact achenes took longer time than those of demucilaged achenes, indicating higher water carrying capacity of intact achenes (Figure 2). The higher capacities for absorbing and carrying water allow the intact achenes to be hydrated for a longer time. However, our study did not measure the dew actually absorbed into the seed embryo cells, and thus future study on how much water is absorbed by the seed embryo and the moisture content (MC) at which repair processes can proceed would be valuable.

It has been proposed that γ-irradiation damage to DNA in the dry embryo is readily repaired on hydration and that the repaired DNA is stable [23]. Our results show that the more hydrated state allows the achene cells to repair their damaged DNA more efficiently before the achenes become dehydrated following sunrise. As a result, a more obvious reduction in DNA damage and much higher DNA repair ratio were observed in irradiated achenes with intact mucilage (Table 1, Figure 4). These results, together with those preliminary results reported in [42], clearly indicate that mucilage provides the achene cells with a more hydrated environment for repairing their damaged DNA during dew deposition and thereby assisting the achene cells in maintenance of their genome integrity.

Rehydration allows repair processes (repair of damaged DNA, proteins, membranes and mitochondria via stored mRNAs) to be activated, and therefore hydration–rehydration cycles can improve seed vigor and germination [51], [52]. Although DNA repair, germination and viability was not synchronic in dew experiment, our data suggest that cumulative DNA repair after 1115 min of dew exposure can effectively maintain the seed germination and viability. The maintenance of germinability would be important for seedling recruitment of this species when suitable conditions for germination occur. Furthermore, our results suggest that achene mucilage hydrated at dew deposition can improve viability of irradiated achenes, which would help the species maintain their soil seed banks. Seed banks are ecologically important components of vegetation dynamics affecting both ecosystem resistance and resilience. In addition, seed banks provide a source of auxiliary recruitment propagules when unfavorable conditions limit flowering and seed production [53]. It has been reported that *A. sphaerocephala* displays highest viable seed numbers in soils on sand dune crest (22.1±9.4/m^−2^) in a 42-year-old stand [54], suggesting the existence of high density seed bank of this species in moving sand dunes. By maintaining the soil seed banks, the effective DNA repair mechanism would facilitate survival and regeneration of this species in the harsh desert environment.

**Figure 5:**
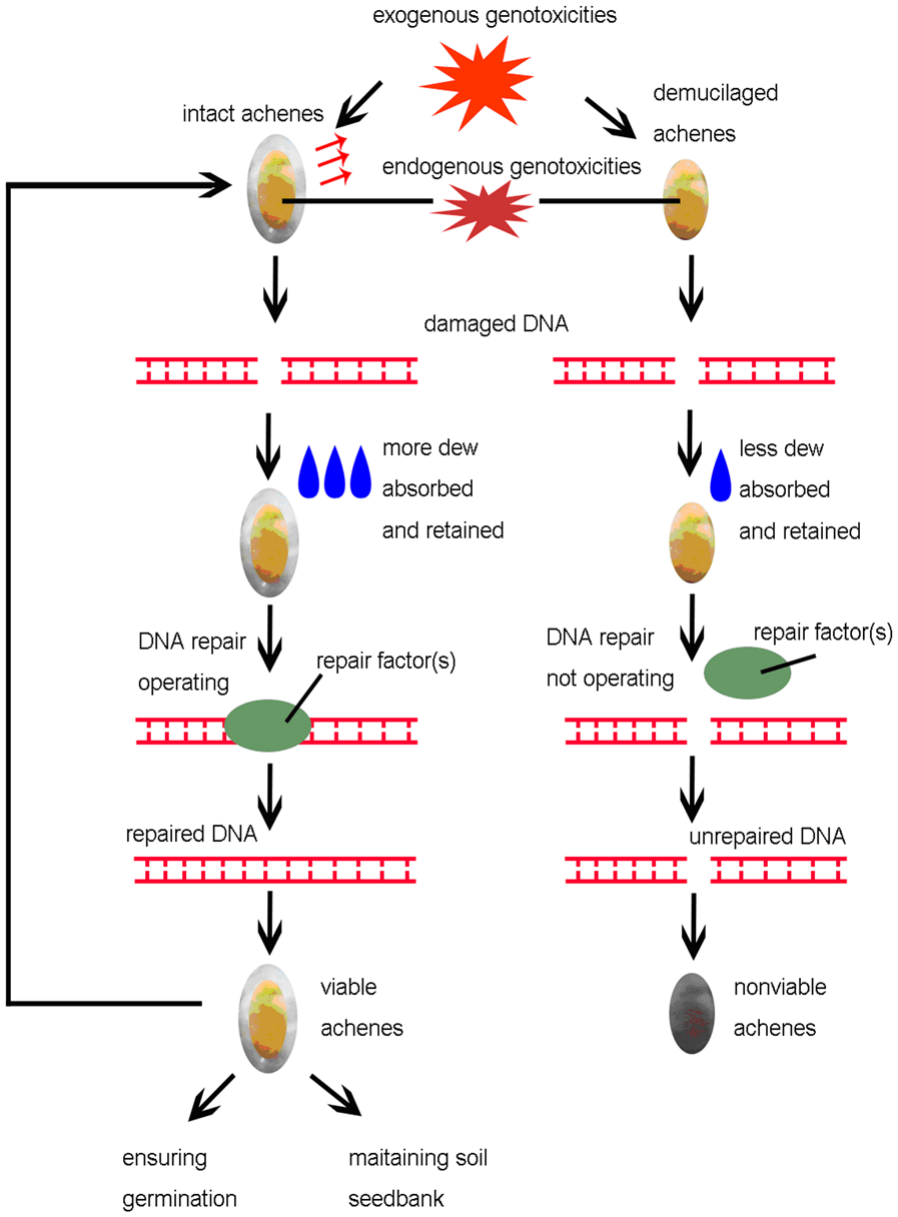
A model for the process of DNA repair of *A. sphaerocephala* achenes, in assistance of mucilage during dew deposition.

Based on the data in this study, we present a model summarizing the processes of DNA repair of *A. sphaerocephala* achenes in assistance of hydrated mucilage during dew deposition (Figure 5). When exposed to exogenous genotoxicities (e.g. high temperature, short periods of moderate seed MC caused by high soil MC at high temperatures and UV light) and endogenous genotoxicities (e.g. cellular oxidative products), DNA damage will occur in the achene cells of *A. sphaerocephala*. During dew deposition, the mucilage enables intact achenes to absorb and retain more water than demucilaged achenes by its high capacities for absorbing and retaining water. Thus, intact achenes are in a more hydrated state, and such a state would initiate the DNA repair factor(s) in achene cells to efficiently repair their DNA damage. As a result, the genome integrity of intact achenes is maintained, and thereby the seed viability is retained. This will in turn ensure achene germination and/or maintain a viable soil seed bank. In contrast, the lack of the mucilage would render demucilaged achenes not effectively initiate the DNA repair factor(s) in the achene cells, thus leading to the loss of seed viability.

In conclusion, this study documents direct evidence that achene mucilage of *A. sphaerocephala* hydrated in desert dew provides the achene cells with a hydrated state for successful repair of cell DNA damage. This long-term survival strategy is vital for this species to survive and regenerate in the desert. Our findings demonstrate that achene mucilage plays an ecologically important role in the life cycle of *A. sphaerocephala* by facilitating DNA repair and maintaining genome integrity of achene cells during dew deposition, and therefore potentially maintaining long-term soil seed banks in genomic-stressful habitats of the harsh desert environment. Future field studies on soil seed bank dynamics of *A. sphaerocephala* are warranted to verify the role of mucilage in the ecological adaptations of this species to its natural habitats. In addition, an artificial soil seed bank can be used to compare the maintenance of seed viability of the two types of achenes in desert environment.

## Materials and Methods

### Ethics approval

The Ethics Review Boards of our institute and the Ordos Sandland Ecological Research Station of the Chinese Academy of Sciences have approved the study protocol.

### Study species

*Artemisia sphaerocephala* Kraschen. (Asteraceae) is one of the most important pioneer plants of the moving and semi-stable sand dunes in the deserts and steppes of northwest and north-central China [55]. This shrub has strong resistance to wind erosion, drought, cold and saline-alkaline soil conditions. *A. sphaerocephala* seeds germinate in spring, and fruits set in August. During maturation, a polysaccharide is secreted through the epidermal cells of the fruit wall and accumulated in layers of mucilage. Involucral bracts of inflorescences protect achenes from wetting by rain until after dispersal [42], [55].

In December 2008, freshly matured achenes of *A. sphaerocephala* were collected from dry unopened infructescences from natural populations near the Ordos Sandland Ecological Research Station of the Chinese Academy of Sciences (39°29′N, 110°11′E; 1296 m a.s.l.) on the Ordos Plateau in Inner Mongolia, north-central China (see a more complete description of this site in [41]). In the laboratory, infructescences were manually shaken to detach the achenes and were stored dry in a closed cotton bag at 5°C and 10% relative humidity until used in experiments.

### Gamma irradiation and mucilage removal

γ-Irradiation has been widely used in DNA damage-dependent cell-cycle profiling studies, survival and check-point activation assays [9]. Thus, batches of intact achenes were treated with ^60^Co γ-irradiation in a ^60^Co γ-AFRRI radiation facility at Institute of Agro-food Science & Technology, Chinese Academy of Agricultural Sciences. For all treatments, a dosage rate of 20 Gy min^−1^ was used. Achenes were treated for 25, 37.5 and 50 min to apply 500, 750 and 1000 Gy of irradiation. After irradiation, the mucilage on the pericarp surface was removed manually from half of the irradiated achenes under a Nikon type 104 projection microscope (Nikon Instruments Inc., Japan). The mucilage was carefully peeled off from the achene coat using a surgical scalpel. Hereafter, the achenes with mucilage removed were defined as “demucilaged achenes”.

### Achene dew exposure treatment in desert

Night-time dew deposition occurs most frequently from July to October in Mu Us Sandland. Dew treatment was conducted near the Ordos Sandland Ecological Research Station of the Chinese Academy of Sciences (39°29′N, 110°11′E; 1296 m a.s.l.). There were four treatments in this experiment: (1) non-irradiated intact achenes (NMA, control); (2) non-irradiated demucilaged achenes (NDA); (3) intact achenes irradiated with 750 Gy (IMA); and (4) demucilaged achenes irradiated with 750 Gy (IDA). Six hundred milligrams of achenes were spread evenly on the surface of sand collected from a nearby field. The sand had been previously rinsed with distilled water and held in Petri dishes to a depth of 10 mm. Petri dishes (4 treatments×5 nights×3 replicates = 60) containing achenes were arranged on trays, and the edges of trays were protected by sticky glue against seed predators, such as ants. Dew experiment was performed three times with similar results, so we only present one experiment here.

At 20:00 pm 28 August, 2009, the trays were brought to the field, and three Petri dishes (replicates) per treatment were sampled every 24 h at 20:00 pm. Intact and demucilaged achenes without irradiation were also sampled as controls. Accumulative dew deposition time was recorded and considered as dew treatment time. Sampled achenes were immediately separated from sand by sieving and were enclosed in Eppendorf tubes until further analysis. To monitor water movement (hydration/dehydration), three replicates of 600 mg intact and demucilaged achenes were also placed on the trays and repeatedly re-weighed every 1 h using an analytical balance (BS221S, Sartorius Group, Germany) throughout the experiment.

Dew experiment was performed for 96 consecutive hours, and no rainfall occurred during the experimental period. Temperature and relative humidity were recorded at 1 min intervals by a Hobo Data Logger (Part No. U23-001, Hobo Pro V2 Temp/RH Data Logger, Onset Computer Corporation, Bourne, MA, USA). Temperature and relative humidity data were processed by Onset HOBOware® Pro software (Version 2.5.0, Onset Computer Corporation, Bourne, MA, USA).

### DNA comet assay of achene cells

Comet assay followed the method described previously with modifications [14], [17]. Briefly, achenes (100 mg) were crushed in liquid nitrogen using pestle and mortar. After transfer into test tubes, the crushed achenes were thoroughly mixed with 3 ml PBS buffer on ice and then filtered through a 100 µm nylon cloth into Eppendorf tubes. Cell suspensions were left at 4°C for 20 min to sediment starch and other fragments. Then 100 µl of supernatant was mixed with 500 µl melted 0.7% LMT agarose (AMRESCO Inc., Solon, OH, USA) at 40°C, and 100 µl of the mixture was immediately pipetted onto a microscope slide, covered with cover slip and then chilled on ice for 1 min to solidify the agarose. After removal of cover slip, slides were immersed in lysis solution (25 g/l SDS in 0.5× TBE buffer) for 2 h and then rinsed in TBE buffer for 5 min. Thereafter, slides were placed into a horizontal electrophoresis chamber with TBE buffer, and electrophoresis was performed at 2 V/cm for 2 min. After rinsing in distilled water for 5 min, slides were stained with 20 µg/ml ethidium bromide for 10 min and then rinsed with distilled water and mounted with a cover slip. Comets were viewed with an inverted fluorescence microscope (Nikon Eclipse Ti, Nikon Instruments Inc., Japan) equipped with a Nikon INTENSILIGHT C-HGFI lamp. TriTek CometScore™ Freeware V 1.5 software (TriTeck Corporation, Sumerduck, VA, USA) was used to evaluate each comet. Comet assays were all performed in triplicate, and 60 comets were analyzed for each slide.

The percentage of cell DNA that migrated into the comet tail (%DNA in tail) was used as a measure of DNA damage. Further, DNA repair ratio was calculated as:


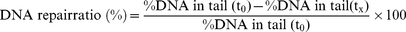


Where %DNA in tail (t_0_) is the DNA damage before dew treatment and %DNA in tail (t_x_) the DNA damage at a given dew treatment time.

### Germination and viability tests

To investigate the effects of dew and mucilage on germinability and viability of the control and irradiated achenes, achenes sampled at different dew treatment times were incubated under germination conditions for 30 d, after which ungerminated achenes were tested for their viability. For each dew treatment time and each of the four treatments (i.e. NMA, NDA, IMA and IDA), three Petri dishes (replicates) with 25 achenes each were used in germination tests. Distilled water (2.5 ml) was added to each Petri dish (5-cm-diameter) with two layers of No. 1 Whatman filter paper, after which they were sealed with Parafilm to minimize water evaporation. The achenes were incubated at a constant temperature of 25°C under continuous fluorescent light (about 100 µmol m^–2^ s^–1^). Germination (radicle emergence) was monitored every 24 h for 30 d, and germination percentages were calculated. Viability of ungerminated achenes after 30-d incubation was tested by the TTC (2,3,5-triphenyl tetrazolium chloride) method [53]. Embryos were placed in 0.5% TTC and incubated at 25°C for 24 h, and then embryos with more than 80% area stained were scored as viable, otherwise they were scored as nonviable.

### Data analysis

A completely randomized design was used in all experiments, and data were expressed as means ± s.e. Proportions were arcsine transformed before statistical analysis to ensure homogeneity of variance, but percentage data shown in table and figures are not transformed. All statistical analysis was performed using SPSS Version 15.0 for Windows (SPSS Inc., Chicago, USA). Two-way ANOVA was used to test the effects of mucilage and dew treatment time on DNA repair ratio and three-way ANOVA to test the effects of irradiation, mucilage and dew treatment time on germination and viability. One-way ANOVA was used to assess the effect of irradiation dose on DNA damage, and independent-samples t-test was performed to detect significant differences (*P*<0.05) between achene types within a treatment.
